# Morphological Clines and Weak Drift along an Urbanization Gradient in the Butterfly, *Pieris rapae*

**DOI:** 10.1371/journal.pone.0083095

**Published:** 2013-12-27

**Authors:** Sean D. Schoville, Ivo Widmer, Magali Deschamps-Cottin, Stéphanie Manel

**Affiliations:** 1 UMR 5525- Laboratoire Techniques de l'Ingénierie Médicale et de la Complexité – Informatique, Mathématiques et Applications, Grenoble, France; 2 UMR 151 – Laboratoire Population Environnement et Développement, Institut de Recherche pour le Développement – Université Aix-Marseille, Marseille, France; 3 UMR 5120- Laboratoire Botanique et BioinforMatique de l'Architecture des Plantes, Montpellier, France; University of Lausanne, Switzerland

## Abstract

Urban areas are increasing globally, providing opportunities for biodiversity researchers to study the process in which species become established in novel, highly disturbed habitats. This ecological process can be understood through analyses of morphological and genetic variation, which can shed light on patterns of neutral and adaptive evolution. Previous studies have shown that urban populations often diverge genetically from non-urban source populations. This could occur due to neutral genetic drift, but an alternative is that selection could lead to allele frequency changes in urban populations. The development of genome scan methods provides an opportunity to investigate these outcomes from samples of genetic variation taken along an urbanization gradient. Here we examine morphological variation in wing size and diversity at neutral amplified fragment length polymorphisms in the butterfly *Pieris rapae* L. (Lepidoptera, Pieridae) sampled from the center to the periphery of Marseille. We utilize established and novel environmental correlation approaches to scan genetic variation for evidence of selection. We find significant morphological differences in urban populations, as well as weak genetic structure and decreased genetic diversity in urban versus non-urban sites. However, environmental correlation tests provide little support for selection in our dataset. Our comparison of different methods and allele frequency clines suggests that loci identified as significant are false positives. Although there is some indication that selection may be acting on wing size in urban butterflies, genetic analyses suggest *P. rapae* are undergoing neutral drift.

## Introduction

Human populations have grown rapidly since 1950, with urban populations increasing nearly five-fold in size at an average rate of 2.6% per year [1]. This dramatic growth has led to massive cityscapes, which pose multifaceted challenges that threaten the survival of local species, largely mirroring trends of global change [2]. These challenges include the increasing fragmentation of natural habitats, novel climatic conditions and environmental hazards, and altered community structure and trophic interactions [3], [4]. Studies of urban areas are thus emerging as important experimental systems in which to investigate how species respond to environmental changes on a recent timescale [5], which is a central goal of evolutionary ecological research.

Perhaps one of the main challenges facing urban species is to maintain viable, interconnected populations when suitable habitats are highly fragmented [6]. Species inhabiting patches of isolated habitat often experience fluctuation in population size, with the continual occupancy of each patch determined by the patch size, extinction probability, and colonization rate [7]. As patches are small in most urban landscapes, a common expectation is that non-urban populations will provide colonists to maintain occupancy in small urban populations [8]. Small populations experience an increase in genetic drift [9], [10], so we would expect to see a decline in genetic variation in urban populations and neutral genetic differentiation from non-urban populations. However, changes in genetic variation are likely to depend on the species mobility [11], as mobile species such as birds exhibit only subtle changes in genetic diversity related to urbanization.

While neutral divergence is one possible response to urbanization, a compelling alternative is that rapid evolutionary adaptation occurs, inducing directional changes in genetic and morphological variation [12]. Rapid evolution can be driven by strong selection on ecological traits, as Badyaev *et al*. [13] demonstrated in documenting the rapid evolution of bill size and courtship song in urban house finches. The relative importance of neutral and adaptive responses to urbanization, and the types of environmental factors that drive adaptive divergence when it occurs, are important questions for urban biodiversity studies. Understanding these factors could improve management strategies that seek to increase species diversity and persistence in urban landscapes [14]. The challenge lies in determining the role of specific environmental factors in generating observed patterns of morphological and genetic variation.

Genome scans are a promising approach to determine whether population genetic patterns result from adaptive genetic changes or genetic drift [15]. One class of genome scans, the *environmental correlation* approach, explicitly tests for correlations between environmental factors and allele frequency distributions [16]. One of the strengths of this approach lies in the individual-based design, which allows for a large number of independent data points to be sampled across a heterogeneous landscape, increasing sensitivity to subtle shifts in allele frequency that might occur as a result of adaptation from standing genetic variation [17]. This approach can be applied to species where population units are difficult to define, as often occurs when gradual changes in genetic variation develop under isolation by distance [17]. Joost et al. [16] proposed using logistic regression to test the effect of each environmental variable. Subsequent implementations of the *environmental correlation* approach have attempted to correct for correlations among individuals due to recent shared ancestry [18], for example by using generalized estimating equations (GEE) to account for relatedness of individuals at nearby sampling locations [19]. We introduce linear mixed-models (LMM) as an alternative to GEE, which allows for among-group variation to be modeled in a nested design and are widely used in genetic association studies [20].

Our study focuses on the butterfly *Pieris rapae* L. (Lepidoptera, Pieridae) sampled near Marseille, to test whether phenotypic changes in wing shape and differences in genetic diversity emerge in urban populations. Urban gradients are known to influence butterfly species diversity [14] and to favor species with certain ecological traits [21], and it has been argued that many urban butterfly populations are small population isolates [22]. However, due to strong selection and because butterfly species often have multiple generations per year, genetic adaptation could occur rapidly. As a result, we test neutral and non-neutral hypotheses to understand the forces that have shaped genetic variability in urban populations of *P. rapae*.

## Materials and Methods

### Ethics Statement

No permits were required for the collection of butterflies in this study and the individuals sampled were killed instantly to minimize suffering.

### Hypothesis Testing

We propose to test hypotheses of drift versus selection in *P. rapae* along an urbanization gradient in Marseille, focusing on two neutral models and one selection model. Under a neutral divergence with gene flow model, morphological differences should not be evident among urban and non-urban sites, genetic diversity should be reduced in urban sites, and changes in genetic structure among urban and non-urban sites should reflect weak genetic drift. Under a neutral geographical isolation model, morphological differences should not be evident among urban and non-urban sites and changes in genetic structure should reflect the isolation of urban populations (i.e. as an abrupt transition in multi-locus allele frequencies), while a decline in genetic diversity is not necessarily expected. Finally, under a model of local adaptation, morphological divergence should be evident in urban sites and a subset of loci should exhibit allele frequency changes that are significantly correlated with changes in environmental variation.

### Sampling and Environmental Data

**Figure 1:**
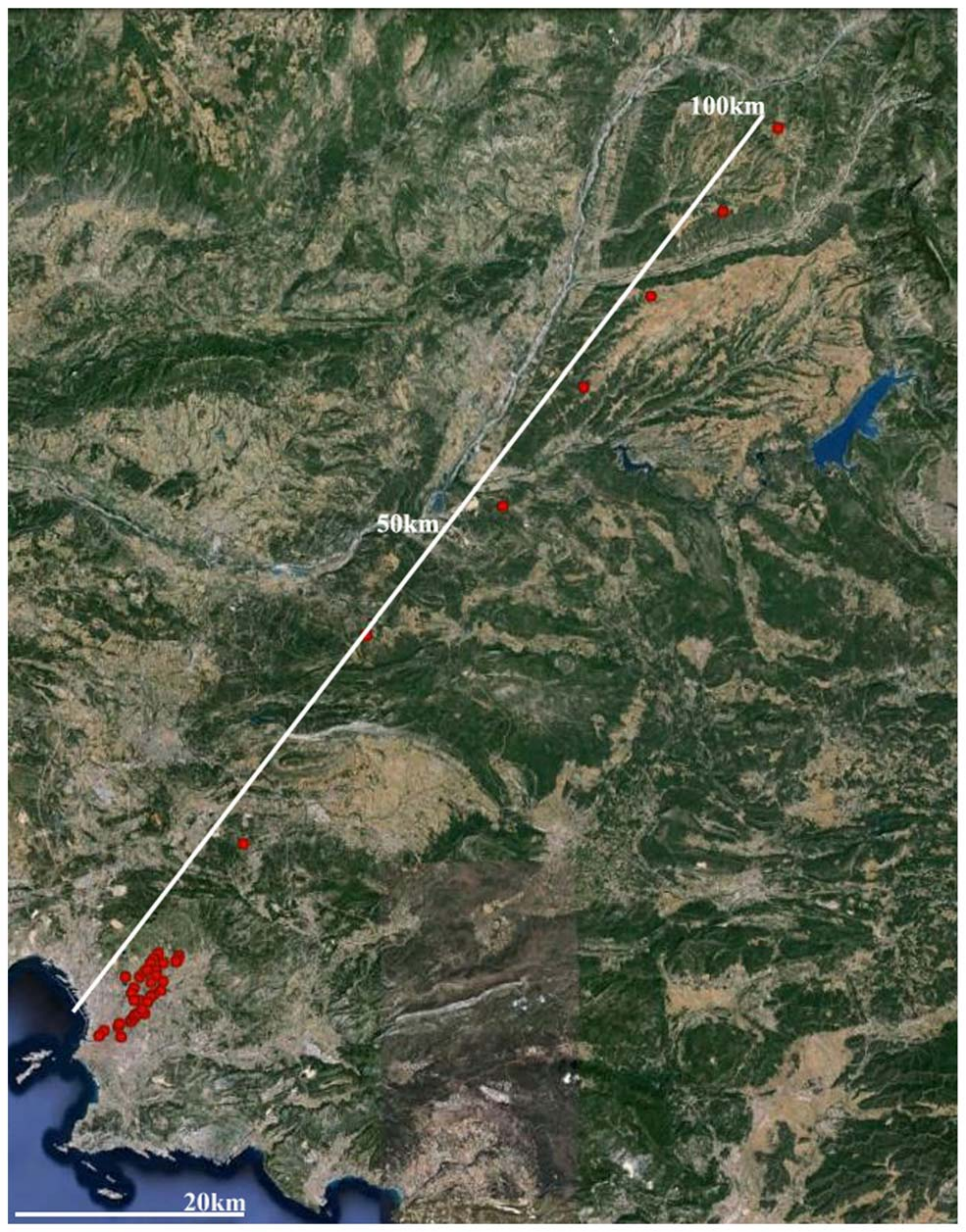
Sampling (red circles) of *Pieris rapae* along a 100 km urbanization gradient in Marseille.

The collection of butterfly samples includes a total of 219 *P. rapae* individuals from 41 sites along a 100 km transect, extending from the center of Marseille northeast through fields and shrubland/forest (**Figure 1**, plotted using Google Earth software). Genetic data consists of 552 AFLP markers amplified from an EcoRI/MseI digest with three primer pairs (EACT-MCAG, EAAC-MCAC and EACT-MCTG). PCR products were labeled with FAM and electrophoresis was performed on a capillary sequencer with a ROX-500 size standard. A panel of 13 individuals was genotyped twice and loci were discarded if errors occurred in more than two individuals. The remaining 366 markers were used in downstream analyses.

We measured wing size variation among the sampled male butterflies, as females represented a small proportion of our total sample size (26%). Six wing size characters were recorded, including forewing length (FL), forewing width (FW), forewing area (FA), hindwing length (HL), hindwing width (HW), and hindwing area (HA; **Figure S1**). Digital images were scanned alongside a metric ruler and measurements were made in Adobe Photoshop CS3 software.

Data on road width (*var1*) were obtained from MapInfo (MapInfo Corporation); in addition, the density and number of buildings in a radius of 150 m were combined as a composite measurement of urbanization (*var2*). Land cover category (*var3*) and percent tree cover (*var4*) were obtained from GlobalMap (NASA Earth Observatory). The free database WorldClim was used to extract 19 climatic variables representing temperature and precipitation averages measured during 1950–2000. To reduce cross-correlations among climatic variables, we identified 16 highly correlated (Pearson's *r>0.8*) WorldClim variables (**Table S1**), combined them in a principal component analysis after standardization, and retained the first two eigenvectors (91.5% and 5.5% of the variation, **Figure S2**) as variables (*var5 & 6*). The three remaining uncorrelated climatic variables, isothermality (*var7*), temperature seasonality (*var8*) and mean diurnal temperature range (*var9*) were analyzed individually.

Genetic, morphological and ecological data have been made publicly available on DRYAD (doi:10.5061/dryad.8837q).

### Analysis of Morphological Variation

We calculated the Pearson correlation coefficient between each morphological variable and the distance of the sampled butterfly from the center of Marseille, and used a *t*-test to assess statistical significance. We also grouped individuals into distance classes, 0–25 km, 25–50 km, 50–75 km, and 75–100 km from the center of Marseille to the periphery, and compared group means for each wing character in an analysis of variance (ANOVA) allowing for unequal variance among groups. We used an *F*-test to assess the significance of differences among group means. The ANOVA was repeated excluding the 25–50 km distance class, due to its smaller sample size. Finally, we calculated the Pearson correlation coefficient between each morphological character and genotypes at each AFLP locus. These correlation tests were corrected for multiple testing using the Dunn-Šidák correction (1– (1– α)1/n), where *n* is the number of variables multiplied by the number of loci (6×366), and the adjusted *p*-value is 2.34×10^–5^. This correction assumes independence among tests and provides a similar, though slightly more conservative correction, than a Bonferroni adjustment.

### Genetic Diversity and Structure

Genetic diversity was measured at each collecting site as the percentage of polymorphic loci and the expected heterozygosity or Nei's gene diversity (*Hj*), using the software Genalex v.6.41 [23]. Using the groups based on distance classes, we measured pairwise *F_ST_* and Nei's genetic distance. These analyses were conducted in the software AFLP-Surv [24], using a Bayesian method [25] developed for biallelic dominant markers [26]. Pairwise *F_ST_* is typically robust to small deviations from demographic equilibrium and provides a measure of genetic differentiation among populations [27]. Finally, all non-urban and urban populations were pooled into two groups to measure pairwise *F_ST_* and Nei's gene diversity.

To examine population structure graphically, we used principal coordinate analysis (PCoA) on all individuals, calculating independent coordinates using a standardized covariance matrix of genetic distance, and plotted the first two coordinates to identify spatial clusters. We then used the Bayesian clustering program Structure v.2.3.3 [28] to assign individuals to populations based on inferred allele frequencies. Using collection sites as prior information, we estimated membership coefficients in one to four clusters (*K*) while allowing for admixture and correlated allele frequencies across populations. We examined 20 runs at each *K* to determine the maximum log-likelihood, setting the burnin to 100,000 and the MCMC sampling to 500,000 steps. Finally, we tested for isolation by distance (IBD) using a Mantel test of the correlation between genetic distance among individuals and Euclidean geographic distance in Genalex. Significance of IBD was tested using 999 permutations of the data.

### Environmental Correlations and Genetic Clines

We used four approaches to test for environmental correlations, including logistic regression [16], generalized estimating equations [19], and two parameterizations of linear mixed models. All models were analyzed in R 2.14.0 (model formula and R code in **Methods S1**). The logistic regression model was implemented using a binomial error distribution and the logit link function, and significance was assessed using an analysis of variance *F*-test: *y_i_*  =  logit-1(*ßX_i_*). The GEE approach was implemented using autocorrelation correlation structure 1 (‘ar1’), which corrects for relatedness among individuals sampled from neighboring localities. The GEE model also uses a binomial error distribution and a Wald test was used to assess significance: *y_i_*  =  logit-1(*ßX_i_*),Var(*y_i_*)  =  *φα_i_*, where the scaling parameter (*φ*) and variance function (*a_i_*) are used to model the covariance structure. For the two linear mixed models, the environmental variable was tested as a fixed effect, while the intercept was allowed to vary across groups. In one version of a LMM (our ‘geographically stratified model’), individuals were grouped into four distance classes defined previously: *y_i_*  =  *ß*_0_ *j*[*i*] + *ß*_1_ *j*[*i*] *X_i_* + *ε_i_*, where fixed effects and intercepts vary for *j* geographical groups. In the second version, the value of each environmental variable (e.g. urbanization, land cover, etc.) at the sample site was used to group individuals into classes (our ‘environmentally stratified model’): *y_i_*  =  *ß*_0_ *j*[*i*] + *ß*_1_ *j*[*i*] *X_i_* + *ε_i_*, where fixed effects and intercepts vary for *j* environmental classifications. Each LMM was implemented with autocorrelation correlation structure 1 (‘ar1’) and the binomial error distribution. The fixed effect of each variable was compared to a null model without the variable using a likelihood-ratio test to assess significance. All environmental correlation tests were corrected for multiple testing using a Dunn-Šidák correction (*n* = 9 variables ×366 loci) with the adjusted *p*-value of 1.56×10^−5^. For loci with significant correlations, we examined allele frequency clines along the Marseille transect using the *hzar* package in R. We plotted and estimated parameters for one-dimensional (1-D) clines, comparing this 1-D model to a null model of frequency changes independent of location using a likelihood ratio test.

## Results

### Morphological Variation

Measurements of the wing size of male butterflies were significantly negatively correlated with distance from the center of Marseille (**Table 1**). Mean values of each wing size character also differed significantly among distance classes in the ANOVA (**Table 1**). These differences remained significant whether the small sample from the 25–50 km distance group was included or not (**Table 1**). On average, wing size characters were larger in male butterflies at urban sites than non-urban sites (**Figure S3)**. Although two loci (14 and 339) were weakly correlated (*r*>0.25) with morphological characters, neither was significant after correction for multiple testing.

**Table 1:** Morphological differences in male wing size along the urbanization gradient, with significant values in bold.

		FL	FW	FA	HL	HW	HA
Correlation with distance from central Marseille	Pearson's *r*	−0.275822	−0.18196	−0.1701	−0.26493	−0.24648	−0.22381
	*p*-value	**0.0005**	**0.0226**	**0.0332**	**0.0008**	**0.0019**	**0.0048**
ANOVA-4 groups	Mean square	32.336	8.056	4552	28.5516	17.945	8760.8
	Residuals	2.335	1.286	834	2.049	1.508	1101.2
	*p*-value	**0.0003**	**0.0133**	**0.0207**	**0.0003**	**0.0007**	**0.0054**
ANOVA-3 groups (without 25–50 km)	Mean square	14.697	7.6739	4336.5	27.465	17.339	8230.7
	Residuals	2.117	1.243	801.8	1.862	1.437	1027.5
	*p*-value	**0.0002**	**0.014**	**0.0214**	**0.0002**	**0.0007**	**0.0053**

### Genetic Diversity and Structure

The percentage of polymorphic loci was high (mean value 31%), with polymorphism increasing from urban to non-urban sites (*r* = 0.573, *p*-value  = 0.0003, **Table S2**). Expected heterozygosity decreased from urban to non-urban samples, but this was largely due to small sample sizes in urban sampling locations. When butterflies were grouped into distance classes, the expected heterozygosity increased from urban sites at 0–25 km (mean 0.10526±0.00638) to non-urban sites at 25–50 km (mean 0.12518±0.00747), 50–75 km (mean 0.12604±0.00687), and 75–100 km (mean 0.11605±0.00668), though this relationship is not significant. Non-urban populations (25–50 km, 50–75 km, and 75–100 km) were not differentiated based on *F_ST_* or Nei's genetic distance (**Table 2**), but these groups were differentiated from urban samples. When all samples were pooled into two groups, *F_ST_* (0.0038) of urban and non-urban samples was statistically significant (*p*-value  = 0.004). Additionally, Nei's gene diversity was significantly higher (*t*-test *p*-value  = 0.0001) in non-urban (0.1617±0.0068) compared to urban populations (0.1457±0.0066).

**Table 2:** Genetic diversity, *F_ST_* (in bold type) and Nei's genetic distance in pooled samples along the urbanization gradient (see Table S2 for non-pooled values).

Distance from central Marseille	Sample Size (N)	Expected heterozygosity (*Hj*)	% Loci Polymorphic	0–25 (km)	25–50 (km)	50–75 (km)	75–100 (km)
0–25km	146	0.105	31.5	-	**0.0036**	**0.0035**	**0.0020**
25–50km	10	0.125	52.2	0.0004	-	**0.0000**	**0.0000**
50–75km	20	0.126	49.5	0.0004	0.0000	-	**0.0000**
75–100km	43	0.116	36.1	0.0002	0.0000	0.0000	-

**Figure 2:**
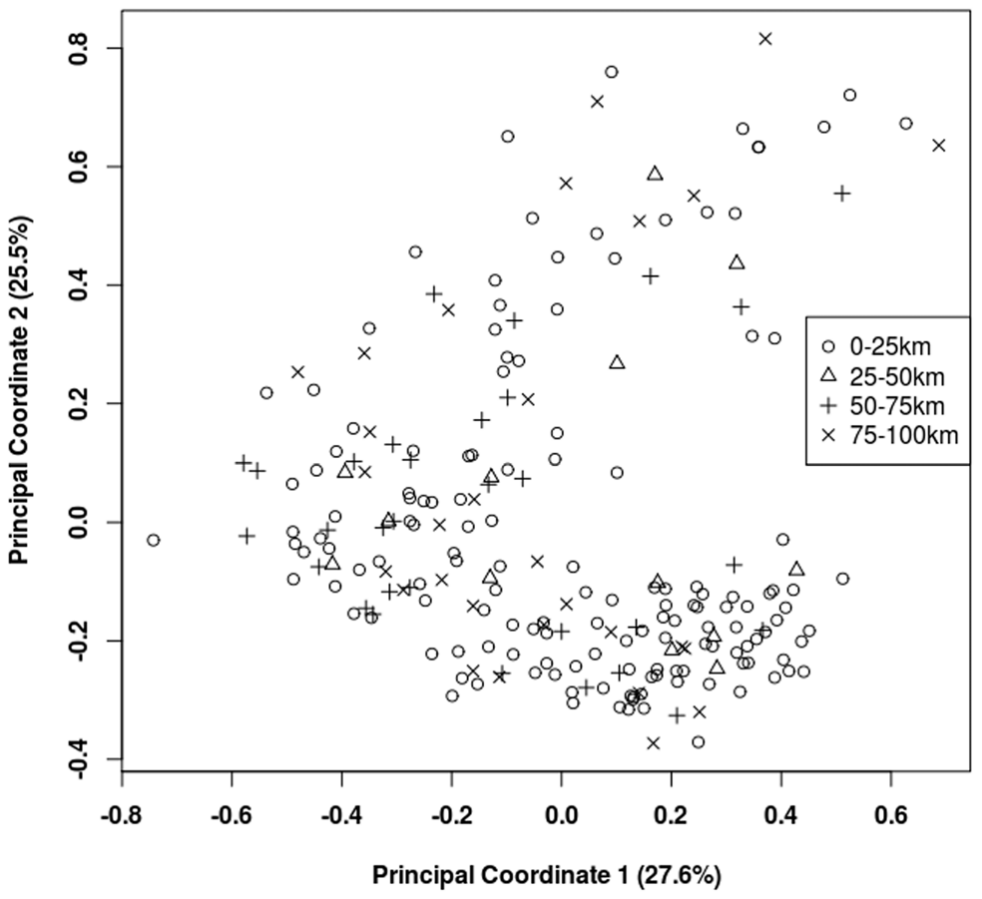
Principal Coordinate Analysis of AFLP variation.

**Figure 3:**
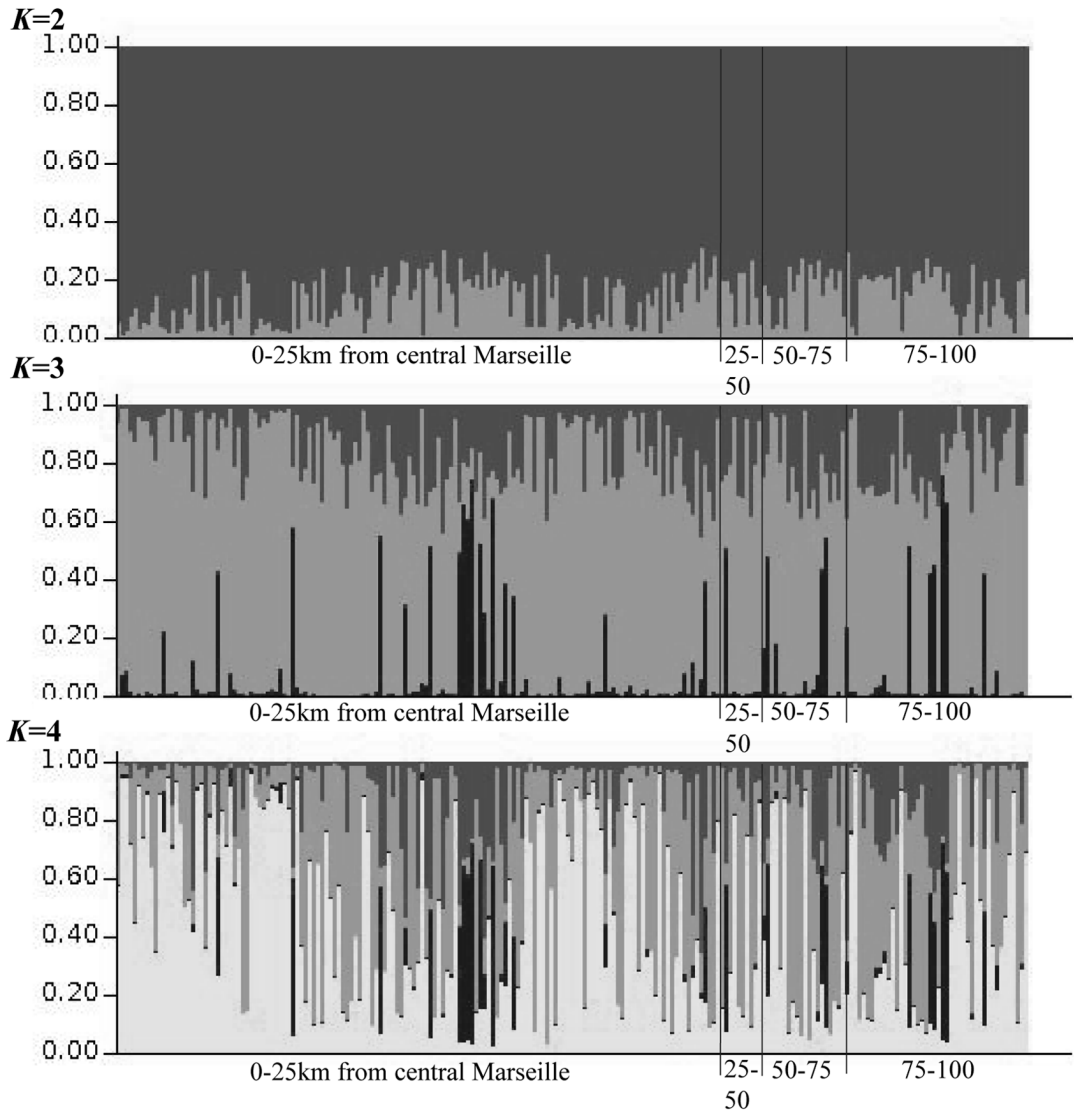
Proportional admixture coefficients of individuals at different levels of *K* clusters based on the Bayesian clustering algorithm Structure. Individuals are ordered left to right according to distance from the center of urban Marseille.

A plot of the first two principal coordinates (27.6% and 25.5% of the variation) provided no evidence of population structure (**Figure 2**). In the Structure analysis, log-likelihood values increased to a maximum at *K* = 4 (*Ln* P(D) = −25839.7). However, individuals were not assigned to discrete clusters when *K*>1 (**Figure 3**), providing no evidence of structure. The IBD test was non-significant (*r* = 0.062, *p*-value  = 0.063).

### Environmental Correlations and Genetic Clines

We found significant correlations using three of the four statistical approaches (**Table 3**). GLM did not detect significant relationships after correction for multiple testing. In contrast, GEE detected 42 significant correlations for 29 loci at seven environmental variables (12 loci were significant at more than one variable). The geographically stratified LMM resulted in six significant tests across three variables, while the environmentally stratified LMM resulted in five significant tests across three variables. Only four loci were significant across more than one statistical model, specifically within the LMM approaches. However, none of the significant loci showed evidence of an allele frequency cline (**Figure S4**), nor were they correlated with morphological characters.

**Table 3:** Loci with significant environmental correlations after correcting for multiple testing (*p-*value <1.56×10^−5^).

	Urbanization	Road Width	% Tree Cover	Land Cover	Isothermality	Temperature Seasonality	Mean Diurnal Temp Range	Temp-Prec PC1	Temp-Prec PC2	Shared across Multiple Variables
**Logistic Regression (GLM)**	None	None	None	None	None	None	None	None	None	None
**Generalized Estimating Equations (GEE)**	88, 126, 130, 223, 334	1, 88, 126, 130, 223	7, 93, 120, 122, 145, 151, 176, 190, 210, 306, 362	7, 56, 93, 112, 120, 122, 128, 145, 156, 168, 176, 190, 208, 210, 239	113, 165, 196	16, 88	None	285	None	**7, 88, 93, 120, 122, 126, 130, 145, 176, 190, 210, 223**
**Linear Mixed Model G (LMMg)**	None	212	47, 225, 281, 288	None	None	None	325	None	None	None
**Linear Mixed Model E (LMMe)**	None	212	225, 281, 288	None	None	None	298	None	None	None
**Shared across Multiple Tests**	None	**212**	**225, 281, 288**	None	None	None	None	None	None	None

## Discussion

Urban environments provide an important experimental system in which to investigate the role of ecological and evolutionary processes as species become established in highly disturbed habitats [12], [29]. While urban environments often lead to relatively homogenous biotic communities [30], this can provide ecological opportunities for successful colonizing species. Urban habitats are particularly well suited to test the relative importance of landscape configuration in driving neutral genetic divergence [31] and ecological gradients in driving adaptive evolution [13]. With the goal of determining whether neutral or adaptive processes influence urban butterfly populations, we examined morphological and genetic changes along an urbanization gradient. The utilization of genome scan methods provides a direct test of whether genetic patterns result from adaptive processes [17].

The butterfly *Pieris rapae* exhibits changes in wing morphology across an urban-to-rural gradient in Marseille (**Table 1**). On average, males have larger wings in urban sites than in non-urban sites, across a variety of measurements including wing length, width and area. Selection could be favoring males that are better dispersers in urban sites, due to the distance among suitable nectar resources and the low population density of potential mates [32]. Large wing size is correlated with dispersal ability and thermal heating rate in butterflies [33] and has been found to be heritable in some *P. rapae* populations [34]. Furthermore, wing size is considered to be a polygenic trait with strong correlations to other fitness-related traits like fecundity and longevity [35]. It is plausible to suggest that urban populations of *P. rapae* are experiencing selection for increased wing size, though our data are not sufficient to exclude alternative neutral hypotheses.

While morphological trends are clear, we found only small differences in genetic variation in a large sample of AFLP markers. Notably, urban butterflies are weakly differentiated from non-urban populations and have a significant decline in diversity, while non-urban populations are not differentiated from one another. The observed genetic differences are not likely to be an artifact of sample size as the pooled urban population consists of a much larger sample of individuals than its non-urban counterpart (**Table 2**). Our observations are consistent with neutral divergence, where declines in variability result from the random effects of drift in a fragmented population [31], [36]. Complete geographical isolation of urban populations is not well supported, as we would expect an abrupt shift in allele frequency across multiple loci, which we do not observe.

One additional possibility is that weak genetic divergence could be explained by selection, if certain alleles are favored in urban relative to non-urban environments. We tested this by examining the correlation between allele frequency changes and environmental variation in urban and non-urban habitats. Our environmental correlation tests included the GLM method [16], as well as the GEE method [19] and LMM methods to account for genetic correlations among sampled individuals. We detected 12 significant AFLP loci (3% of all loci) using the GEE method and none using GLM. Seven loci were detected in the LMM approaches. Only four loci were significant in multiple methods, all under the LMM models. However, these LMM models are quite similar in design, and their concordance is not entirely unexpected. The lack of shared significant loci across the other methods suggests that we might be detecting false positives. Furthermore, the analyses of allele frequency clines provide no evidence for geographical transitions in frequency along the urbanization gradient. Similarly, tests for associations of allele frequencies with wing size traits were not significant.

Recent simulation studies suggest that environmental correlation tests might suffer from a high rate of false positives [37], particularly when the underlying genetic structure is not modeled appropriately by the covariance matrix. While the intent of the GEE and LMM methods was to correct for underlying genetic correlations among sampled individuals, the subtle change in genetic structure might not be captured by these models. One solution to this problem is to evaluate the environmental correlation tests based on the output of multiple statistical models. In our dataset, we find little concordance among the different methods. We conclude that selection does not account for the observed genetic differences, but we must add the caveat that our sampling reflects only a small fraction of the *P. rapae* genome (expected coverage <0.1% for a typical butterfly genome of 400 Mb). Given the observed shifts in morphological traits, it is possible that evidence of adaptation might be found with more comprehensive coverage. The recent developments in next generation sequencing in non-model organisms [38] should increase genome coverage and improve future efforts to detect genetic adaptation in *P. rapae*.

Numerous studies have focused on how biodiversity and ecological traits change along urban gradients [12], [29], showing that urbanization favors highly dispersive generalists [39]. Comparatively little is known, however, about whether genetic changes occur when organisms are established in urban landscapes [31]. Here we show that butterfly populations in urban Marseille have diverged morphologically and genetically. Selection might favor larger sized male butterflies, whereas the observed genetic variation is consistent with neutral genetic drift. Future efforts will need to expand genetic sampling and incorporate quantitative genetic analyses of resident urban and non-urban populations to determine if morphological differences in urban *P. rapae* are heritable and under selection.

## Supporting Information

Figure S1 **Measurements of wing size taken on male butterflies. FL  =  forewing length, FW  =  forewing width, HL  =  hindwing length, HW  =  hindwing width.**
(PDF)

Figure S2 **Principal component analysis (PCA) of temperature and precipitation variables at the sample sites.**
(PDF)

Figure S3 **Boxplots of morphological variation in male butterflies taken at distances measured from the center of Marseille along a gradient of urbanization.**
(PDF)

Figure S4 **Plots of the allele frequency clines of significant loci as a function of distance (meters) from Marseille.**
(PDF)

Table S1 **Pearson correlations among variables, showing in bold climatic variables with significant correlations (** ***r*** **) greater than 0.80.**
(PDF)

Table S2 **Summary of AFLP genetic variation at each sampling site.**
(PDF)

Methods S1 **Statistical model formulas and details of implementation in the computing environment R.**
(PDF)

## References

[1] United Nations (2012) World Urbanization Prospects, the 2011 Revision. New York: Department of Economic and Social Affairs, Population Division.

[2] Grimm NB, Faeth SH, Golubiewski NE, Redman CL, Wu J, et al (2008) Global change and the ecology of cities. Science 319: 756–760. 18258902 10.1126/science.1150195

[3] McDonnell MJ, Pickett STA, Groffman P, Bohlen P, Pouyat RV, et al (1997) Ecosystem processes along an urban-to-rural gradient. Urban Ecosystems 1: 21–36.

[4] Dearborn DC, Kark S (2009) Motivations for conserving urban biodiversity. Conserv Biol 24: 432–440. 19775276 10.1111/j.1523-1739.2009.01328.x

[5] Manel S, Holderegger R (2013) Ten years of landscape genetics. Trends Ecol Evol 28: 614–621. 23769416 10.1016/j.tree.2013.05.012

[6] Carreiro MM, Tripler CE (2005) Forest remnants along urban-rural gradients: examining their potential for global change research. Ecosystems 8: 568–582.

[7] Moilanen A, Hanski I (1998) Metapopulation dynamics: effects of habitat quality and landscape structure. Ecology 79: 2503–2515.

[8] Stracey CM, Robinson SK (2012) Are urban habitats ecological traps for a native songbird? Season-long productivity, apparent survival, and site fidelity in urban and rural habitats. J Avian Biol 43: 50–60.

[9] Young A, Boyle T, Brown T (1996) The population genetic consequences of habitat fragmentation for plants. Trends Ecol Evol 11: 413–418. 21237900 10.1016/0169-5347(96)10045-8

[10] Gaggiotti OE (1996) Population genetic models of source-sink metapopulations. Theor Popul Biol 50: 178–208. 8955032 10.1006/tpbi.1996.0028

[11] Unfried TM, Hauser L, Marzluff JM (2013) Effects of urbanization on Song Sparrow (*Melospiza melodia*) population connectivity. Conserv Genet 14: 41–53.

[12] Francis RA, Chadwick MA (2012) What makes a species synurbic? Appl Geogr 32: 514–521.

[13] Badyaev AV, Young RL, Oh KP, Addison C (2008) Evolution on a local scale: Developmental, functional, and genetic bases of divergence in bill form and associated changes in song structure between adjacent habitats. Evolution 62: 1951–1964. 18507745 10.1111/j.1558-5646.2008.00428.x

[14] Lizee MH, Manel S, Mauffrey JF, Tatoni T, Deschamps-Cottin M (2012) Matrix configuration and patch isolation influences override the species-area relationship for urban butterfly communities. Landscape Ecol 27: 159–169.

[15] Storz JF (2005) Using genome scans of DNA polymorphism to infer adaptive population divergence. Mol Ecol 14: 671–688. 15723660 10.1111/j.1365-294X.2005.02437.x

[16] Joost S, Bonin A, Bruford MW, Després L, Conord C, et al (2007) A spatial analysis method (SAM) to detect candidate loci for selection: towards a landscape genomics approach to adaptation. Mol Ecol 16: 3955–3969. 17850556 10.1111/j.1365-294X.2007.03442.x

[17] Schoville SD, Bonin A, François O, Lobreaux S, Melodelima C, et al (2012) Adaptive genetic variation on the landscape: methods and cases. Annu Rev Ecol, Evol Syst 43: 23–43.

[18] Manel S, Joost S, Epperson B, Storfer A, Holderegger R, et al (2010) Perspectives on the use of landscape genetics to detect genetic adaptive variation in the field. Mol Ecol 19: 3760–3772. 20723056 10.1111/j.1365-294X.2010.04717.x

[19] Poncet BN, Herrmann D, Gugerli F, Taberlet P, Holderegger R, et al (2010) Tracking genes of ecological relevance using a genome scan in two independent regional population samples of *Arabis alpina* . Mol Ecol 19: 2896–2907. 20609082 10.1111/j.1365-294X.2010.04696.x

[20] Zhang Z, Ersoz E, Lai C-Q, Todhunter RJ, Tiwari HK, et al (2010) Mixed linear model approach adapted for genome-wide association studies. Nat Genet 42: 355–360. 20208535 10.1038/ng.546 PMC2931336

[21] McIntyre NE (2000) Ecology of urban arthropods: A review and a call to action. Ann Entomol Soc Am 93: 825–835.

[22] Altermatt F (2012) Temperature-related shifts in butterfly phenology depend on the habitat. Global Change Biol 18: 2429–2438.

[23] Peakall R, Smouse PE (2006) GENALEX 6: genetic analysis in Excel. Population genetic software for teaching and research. Mol Ecol Notes 6: 288–295. 10.1093/bioinformatics/bts460 PMC3463245 22820204

[24] Vekemans X (2002) AFLP-SURV 1.0: A program for genetic diversity analysis with AFLP (and RAPD) population data. Laboratoire de Génétique et d'Ecologie Végétales, Université Libre de Bruxelles.

[25] Zhivotovsky LA (1999) Estimating population structure in diploids with multilocus dominant DNA markers. Mol Ecol 8: 907–913. 10434412 10.1046/j.1365-294x.1999.00620.x

[26] Lynch M, Milligan B (1994) Analysis of population genetic structure with RAPD markers. Mol Ecol 3: 91–99. 8019690 10.1111/j.1365-294x.1994.tb00109.x

[27] Beaumont MA (2005) Adaptation and speciation: what can *F_ST_* tell us? Trends Ecol Evol 20: 435–440. 16701414 10.1016/j.tree.2005.05.017

[28] Pritchard JK, Stephens M, Donnelly P (2000) Inference of population structure using multilocus genotype data. Genetics 155: 945–959. 10835412 10.1093/genetics/155.2.945 PMC1461096

[29] Pickett STA, Cadenasso ML, Grove JM, Boone CG, Groffman PM, et al (2011) Urban ecological systems: Scientific foundations and a decade of progress. J Environ Manage 92: 331–362. 20965643 10.1016/j.jenvman.2010.08.022

[30] McKinney ML (2006) Urbanization as a major cause of biotic homogenization. Biol Conserv 127: 247–260.

[31] Delaney KS, Riley SPD, Fisher RN (2010) A rapid, strong, and convergent genetic response to urban habitat fragmentation in four divergent and widespread vertebrates. PLoS One 5: e12767. 20862274 10.1371/journal.pone.0012767 PMC2940822

[32] Sekar S (2012) A meta-analysis of the traits affecting dispersal ability in butterflies: can wingspan be used as a proxy? J Anim Ecol 81: 174–184. 21988561 10.1111/j.1365-2656.2011.01909.x

[33] Ducatez S, Baguette M, Trochet A, Chaput-Bardy A, Legrand D, et al.. (2012) Flight endurance and heating rate vary with both latitude and habitat connectivity in a butterfly species. Oikos.

[34] Tanaka Y (1987) Polygenic analyses of morphological characters in *Pieris rapae crucivora* Boisduval (Lepidoptera: Pieridae). I. Heritability estimates. Appl Entomol Zool 22: 125–132.

[35] Kimura K, Tsubaki Y (1986) Female size and age-specific fecundity in the small white butterfly, *Pieris rapae crucivora* Boisduval (Lepidoptera: Pieriae). Res Popul Ecol 28: 295–304.

[36] Andreasen AM, Stewart KM, Longland WS, Beckmann JP, Forister ML (2012) Identification of source-sink dynamics in mountain lions of the Great Basin. Mol Ecol 21: 5689–5701. 22934825 10.1111/j.1365-294X.2012.05740.x

[37] De Mita S, Thuillet A-C, Gay L, Ahmadi N, Manel S, et al (2013) Detecting selection along environmental gradients: analysis of eight methods and their effectiveness for outbreeding and selfing populations. Mol Ecol 22: 1383–1399. 23294205 10.1111/mec.12182

[38] Ekblom R, Galindo J (2010) Applications of next generation sequencing in molecular ecology of non-model organisms. Heredity 107: 1–15. 21139633 10.1038/hdy.2010.152 PMC3186121

[39] Clergeau P, Croci S, Jokimäki J, Kaisanlahti-Jokimäki M-L, Dinetti M (2006) Avifauna homogenisation by urbanisation: Analysis at different European latitudes. Biol Conserv 127: 336–344.

