# Correction: Morphological Clines and Weak Drift along an Urbanization Gradient in the Butterfly, *Pieris rapae*

**DOI:** 10.1371/journal.pone.0096685

**Published:** 2014-04-29

**Authors:** The PLOS ONE Staff

Several authors are included in the Acknowledgements instead of the author byline. The following individuals should be listed as authors:

Marie-Hélène Lizée^1^ should be listed as the fourth author. The contributions of this author are as follows: performed the experiments.

Laurence Després^2^ should be listed as the fifth author. The contributions of this author are as follows: Conceived and designed the experiments, Contributed reagents/materials/analysis tools.

Delphine Rioux^2^ should be listed as the sixth author. The contributions of this author are as follows: Performed the experiments.

Ludovic Gielly^2^ should be listed as the seventh author. The contributions of this author are as follows: Performed the experiments.

1 UMR 151 - Laboratoire Population Environnement et Développement, Institut de Recherche pour le Développement - Université Aix-Marseille, Marseille, France

2 Laboratoire d'Ecologie Alpine, Université Grenoble Alpes, CNRS, Grenoble, France

The correct citation is: Schoville SD, Widmer I, Deschamps-Cottin M, Lizée M, Després L (2013) Morphological Clines and Weak Drift along an Urbanization Gradient in the Butterfly, *Pieris rapae*. PLoS ONE 8(12): e83095. doi:10.1371/journal.pone.0083095
