# Supplementary material for: Morphological Clines and Weak Drift along an Urbanization Gradient in the Butterfly, Pieris rapae
Source: PLoS One. 2013 Dec 27;8(12):e83095. doi: 10.1371/journal.pone.0083095 (PMC3873920; doi:10.1371/journal.pone.0083095)
Supplement: Figure S1 — Measurements of wing size taken on male butterflies. FL = forewing length, FW = forewing width, HL = hindwing length, HW = hindwing width. (PDF) [file pone.0083095.s001.pdf]

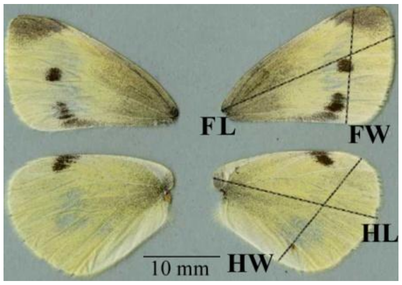

Figure S1. Measurements of wing size taken on male butterflies. FL = forewing length, FW = forewing width, HL = hindwing length, HW = hindwing width.
