# Supplementary material for: Morphological Clines and Weak Drift along an Urbanization Gradient in the Butterfly, Pieris rapae
Source: PLoS One. 2013 Dec 27;8(12):e83095. doi: 10.1371/journal.pone.0083095 (PMC3873920; doi:10.1371/journal.pone.0083095)
Supplement: Figure S2 — Principal component analysis (PCA) of temperature and precipitation variables at the sample sites. (PDF) [file pone.0083095.s002.pdf]

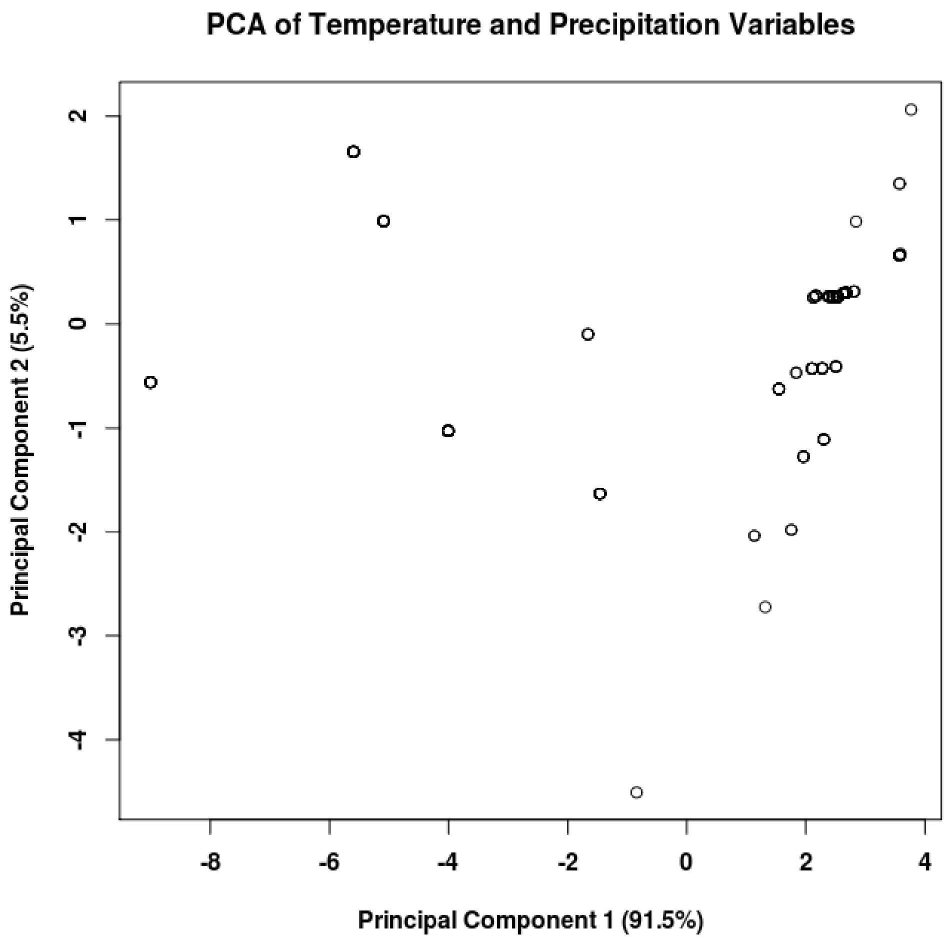

Figure S2. Principal component analysis (PCA) of temperature and precipitation variables at the sample sites.
