# Supplementary material for: Morphological Clines and Weak Drift along an Urbanization Gradient in the Butterfly, Pieris rapae
Source: PLoS One. 2013 Dec 27;8(12):e83095. doi: 10.1371/journal.pone.0083095 (PMC3873920; doi:10.1371/journal.pone.0083095)
Supplement: Figure S3 — Boxplots of morphological variation in male butterflies taken at distances measured from the center of Marseille along a gradient of urbanization. (PDF) [file pone.0083095.s003.pdf]

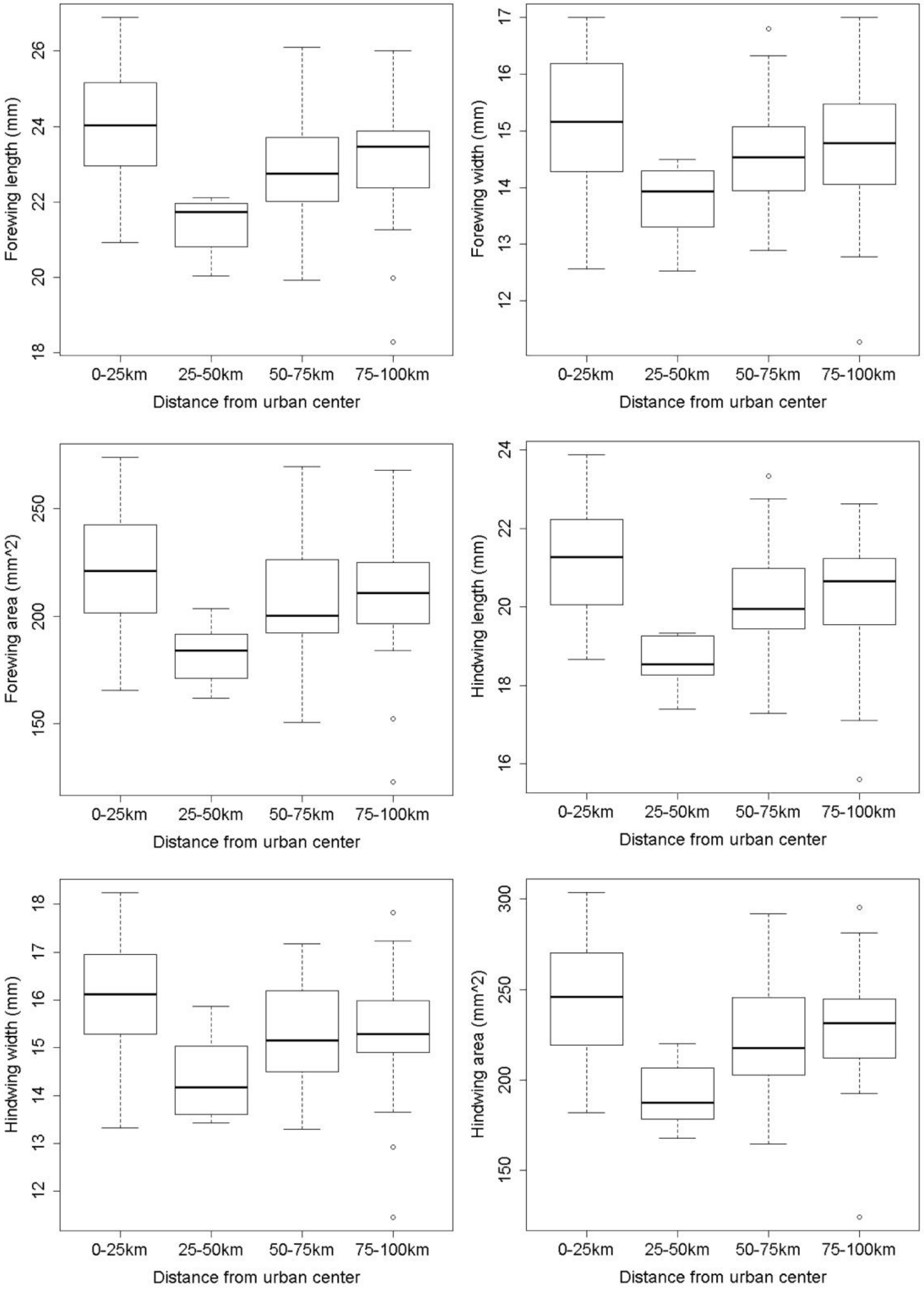

Figure S3. Boxplots of morphological variation in male butterflies taken at distances measured from the center of Marseille along a gradient of urbanization.
