# Supplementary material for: Morphological Clines and Weak Drift along an Urbanization Gradient in the Butterfly, Pieris rapae
Source: PLoS One. 2013 Dec 27;8(12):e83095. doi: 10.1371/journal.pone.0083095 (PMC3873920; doi:10.1371/journal.pone.0083095)
Supplement: Figure S4 — Plots of the allele frequency clines of significant loci as a function of distance (meters) from Marseille. (PDF) [file pone.0083095.s004.pdf]

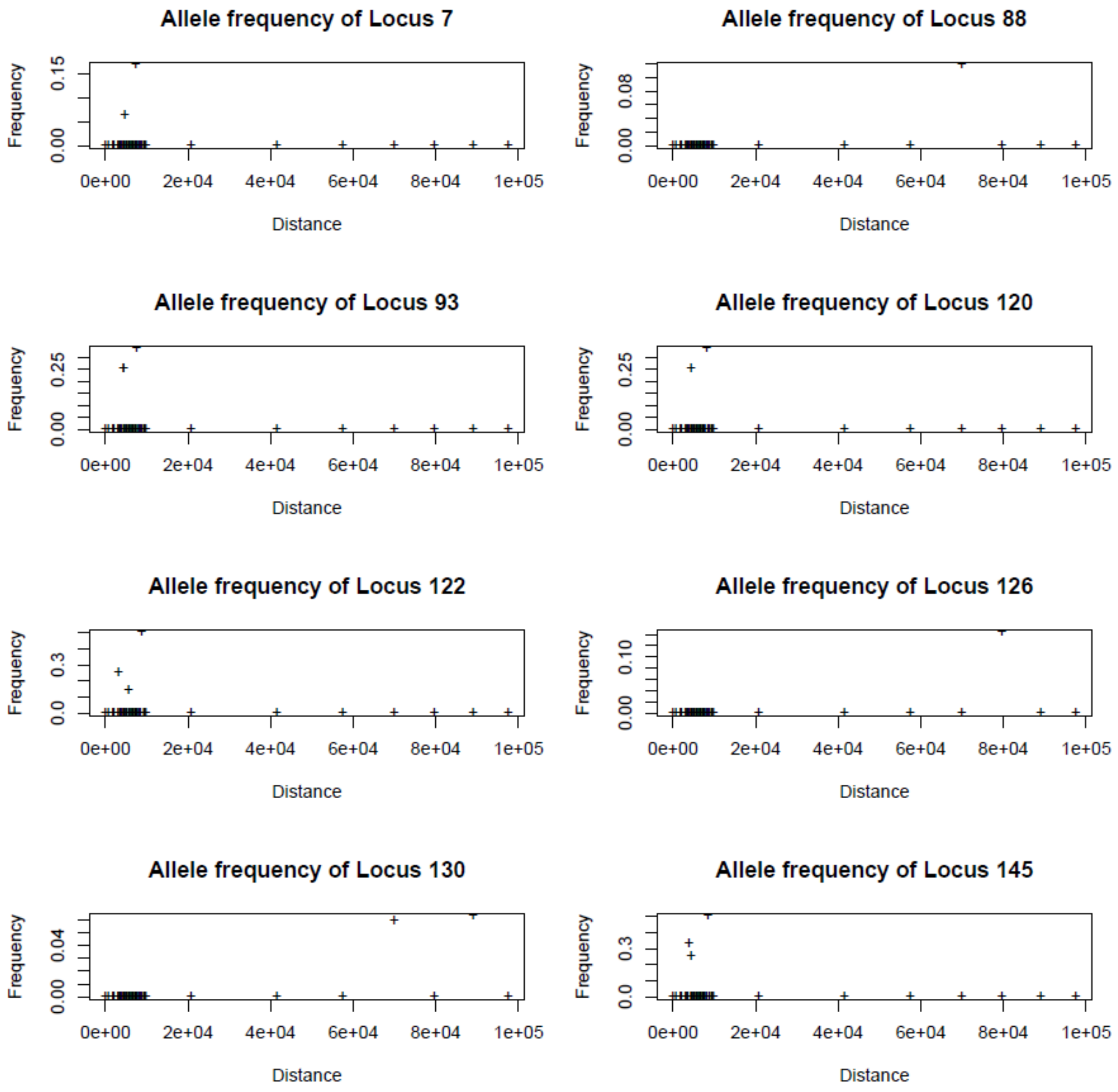
Allele frequency of Locus 7
Frequency
0.00
0.15
0e+00
2e+04
4e+04
6e+04
8e+04
1e+05
Distance
Allele frequency of Locus 88
Frequency
0.00
0.08
Distance
Allele frequency of Locus 93
Frequency
0.00
0.25
Distance
Allele frequency of Locus 120
Frequency
0.00
0.25
Distance
Allele frequency of Locus 122
Frequency
0.0
0.3
Distance
Allele frequency of Locus 126
Frequency
0.00
0.10
Distance
Allele frequency of Locus 130
Frequency
0.00
0.04
Distance
Allele frequency of Locus 145
Frequency
0.0
0.3
Distance

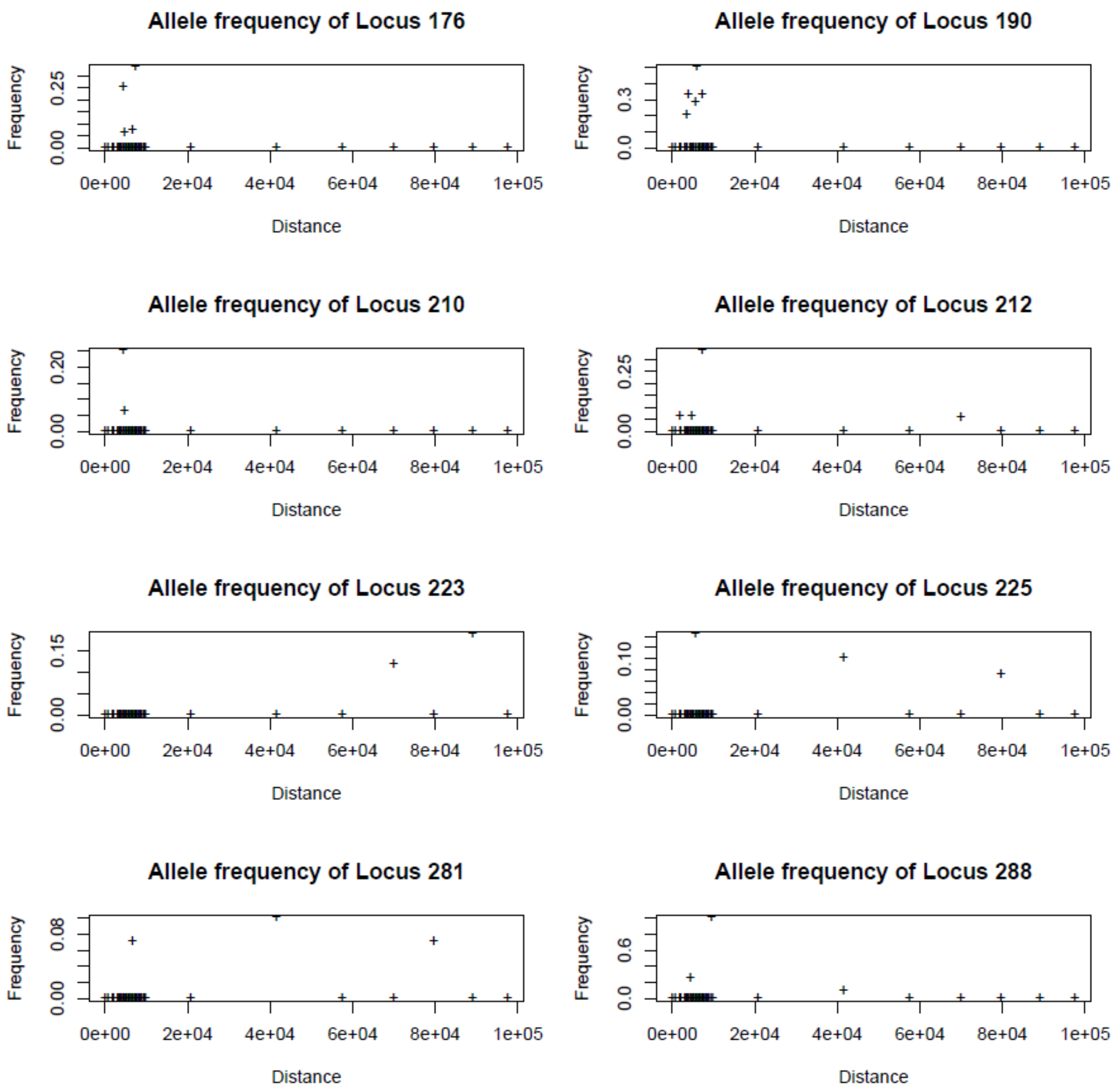

Figure S4. Plots of the allele frequency clines of significant loci as a function of distance (meters) from Marseille.
