# Supplementary material for: Morphological Clines and Weak Drift along an Urbanization Gradient in the Butterfly, Pieris rapae
Source: PLoS One. 2013 Dec 27;8(12):e83095. doi: 10.1371/journal.pone.0083095 (PMC3873920; doi:10.1371/journal.pone.0083095)
Supplement: Table S1 — Pearson correlations among variables, showing in bold climatic variables with significant correlations ( r ) greater than 0.80. (PDF) [file pone.0083095.s005.pdf]

Table S1. Pearson correlations among variables, showing in bold climatic variables with significant correlations (*r*) greater than 0.80.

	**1**	**2**	**3**	**4**	**5**	**6**	**7**	**8**	**9**	**10**	**11**	**12**	**13**	**14**	**15**	**16**	**17**	**18**	**19**	**20**	**21**	**22**	**23**	**24**	**25**
**1** Annual mean temperature	1.00																								
**2** Mean diurnal range	-0.75	1.00																							
**3** Isothermality	-0.30	0.45	1.00																						
**4** Temperature seasonality	0.21	0.06	0.02	1.00																					
**5** Max temperature warmest month	**1.00**	-0.74	-0.32	0.24	1.00																				
**6** Min temperature coldest month	**1.00**	-0.78	-0.31	0.17	**1.00**	1.00																			
**7** Temperature annual range	-0.44	0.76	0.03	0.53	-0.41	-0.49	1.00																		
**8** Mean temperature wettest quarter	**0.83**	-0.51	-0.11	0.32	**0.83**	**0.82**	-0.27	1.00																	
**9** Mean temperature driest quarter	**1.00**	-0.75	-0.30	0.23	**1.00**	**1.00**	-0.43	**0.83**	1.00																
**10** Mean temperature warmest quarter	**1.00**	-0.75	-0.30	0.23	**1.00**	**1.00**	-0.43	**0.83**	**1.00**	1.00															
**11** Mean temperature coldest quarter	**1.00**	-0.77	-0.31	0.18	**1.00**	**1.00**	-0.47	**0.82**	**1.00**	**1.00**	1.00														
**12** Annual precipitation	**-1.00**	0.78	0.31	-0.26	**-1.00**	**-0.99**	0.44	**-0.82**	**-1.00**	**-1.00**	**-0.99**	1.00													
**13** Precipitation wettest month	**-0.92**	0.53	0.27	-0.15	**-0.93**	**-0.91**	0.25	-0.73	**-0.92**	**-0.92**	**-0.92**	**0.90**	1.00												
**14** Precipitation driest month	**-1.00**	**0.80**	0.31	-0.19	**-0.99**	**-1.00**	0.49	**-0.81**	**-1.00**	**-1.00**	**-1.00**	**1.00**	**0.89**	1.00											
**15** Precipitation seasonality	**0.98**	**-0.82**	-0.32	0.30	**0.98**	**0.98**	-0.45	**0.82**	**0.98**	**0.98**	**0.98**	**-0.99**	**-0.84**	**-0.99**	1.00										
**16** Precipitation wettest quarter	**-0.99**	0.69	0.29	-0.27	**-1.00**	**-0.99**	0.35	**-0.84**	**-0.99**	**-0.99**	**-0.99**	**0.99**	**0.95**	**0.98**	**-0.97**	1.00									
**17** Precipitation driest quarter	**-0.99**	**0.81**	0.31	-0.22	**-0.99**	**-1.00**	0.48	**-0.81**	**-1.00**	**-1.00**	**-0.99**	**1.00**	**0.89**	**1.00**	**-0.99**	**0.98**	1.00								
**18** Precipitation warmest quarter	**-0.99**	**0.81**	0.31	-0.22	**-0.99**	**-1.00**	0.48	**-0.81**	**-1.00**	**-1.00**	**-0.99**	**1.00**	**0.89**	**1.00**	**-0.99**	**0.98**	**1.00**	1.00							
**19** Precipitation coldest quarter	**-0.99**	0.72	0.29	-0.34	**-0.99**	**-0.98**	0.35	**-0.84**	**-0.99**	**-0.99**	**-0.99**	**0.99**	**0.91**	**0.98**	**-0.98**	**0.99**	**0.99**	**0.99**	1.00						
**20** Percent tree cover	-0.11	0.28	0.13	-0.04	-0.10	-0.11	0.18	0.08	-0.11	-0.11	-0.11	0.12	0.07	0.13	-0.14	0.10	0.13	0.13	0.11	1.00					
**21** Land cover type	0.83	-0.75	-0.29	0.50	0.83	0.82	-0.28	0.68	0.84	0.84	0.82	-0.87	-0.67	-0.84	0.90	-0.83	-0.86	-0.86	-0.87	-0.23	1.00				
**22** Building area (m^2)	0.66	-0.41	-0.28	0.48	0.67	0.64	-0.03	0.74	0.67	0.67	0.65	-0.68	-0.55	-0.65	0.70	-0.68	-0.66	-0.66	-0.71	0.10	0.64	1.00			
**23** Building density	0.66	-0.41	-0.29	0.48	0.67	0.65	-0.02	0.74	0.67	0.67	0.65	-0.68	-0.56	-0.65	0.70	-0.68	-0.66	-0.66	-0.71	0.09	0.64	1.00	1.00		
**24** Road width (m)	0.67	-0.55	-0.35	0.29	0.67	0.67	-0.23	0.52	0.68	0.68	0.66	-0.68	-0.55	-0.67	0.71	-0.66	-0.68	-0.68	-0.69	-0.10	0.62	0.77	0.77	1.00	
**25** Urbanization	0.76	-0.47	-0.21	0.52	0.77	0.75	-0.10	0.89	0.77	0.77	0.75	-0.78	-0.64	-0.75	0.80	-0.78	-0.76	-0.76	-0.81	0.01	0.75	0.91	0.91	0.67	1.00
