# Supplementary material for: Morphological Clines and Weak Drift along an Urbanization Gradient in the Butterfly, Pieris rapae
Source: PLoS One. 2013 Dec 27;8(12):e83095. doi: 10.1371/journal.pone.0083095 (PMC3873920; doi:10.1371/journal.pone.0083095)
Supplement: Table S2 — Summary of AFLP genetic variation at each sampling site. (PDF) [file pone.0083095.s006.pdf]

**Table S2**. Summary of AFLP genetic variation at each sampling site.

| Site No | Latitude | Longitude | Distance Class | Location Name | Sample Size (N) | % Loci Polymorphic | Expected Heterozygosity (Hj) |
|---|---|---|---|---|---|---|---|
| 1 | 43.30162 | 5.37997 | 0-25km | Saint Charles | 1 | N/A | N/A |
| 2 | 43.29684 | 5.39801 | 0-25km | Georges | 2 | 4.62% | 0.34062 (±0.00347) |
| 3 | 43.30704 | 5.39623 | 0-25km | Ricarc Digne | 1 | N/A | N/A |
| 4 | 43.30863 | 5.40806 | 0-25km | Chenil | 1 | N/A | N/A |
| 5 | 43.30999 | 5.40907 | 0-25km | MontolivetC | 4 | 16.30% | 0.24567 (±0.00515) |
| 6 | 43.31187 | 5.41252 | 0-25km | Gillet | 5 | 22.83% | 0.2215 (±0.00542) |
| 7 | 43.31368 | 5.41289 | 0-25km | MontolivetB | 4 | 20.92% | 0.25774 (±0.00565) |
| 8 | 43.31519 | 5.41604 | 0-25km | Corbière | 3 | 20.11% | 0.29736 (±0.0051) |
| 9 | 43.31829 | 5.42040 | 0-25km | MontolivetA | 4 | 26.09% | 0.26067 (±0.00549) |
| 10 | 43.32514 | 5.41232 | 0-25km | Bogue | 4 | 19.84% | 0.25278 (±0.00556) |
| 11 | 43.33046 | 5.40933 | 0-25km | Allende | 4 | 26.36% | 0.26687 (±0.00584) |
| 12 | 43.32379 | 5.42616 | 0-25km | Frais Vallon | 4 | 15.49% | 0.24664 (±0.00533) |
| 13 | 43.33476 | 5.41202 | 0-25km | Polytres | 2 | 8.15% | 0.34699 (±0.00389) |
| 14 | 43.32843 | 5.42974 | 0-25km | Sartre | 4 | 20.65% | 0.24732 (±0.00526) |
| 15 | 43.32996 | 5.43268 | 0-25km | Bengalis | 4 | 24.18% | 0.26224 (±0.00581) |
| 16 | 43.34348 | 5.41807 | 0-25km | Mitre | 4 | 24.18% | 0.26322 (±0.00558) |
| 17 | 43.33910 | 5.43024 | 0-25km | Consolation | 4 | 20.65% | 0.25846 (±0.0057) |
| 18 | 43.34685 | 5.42297 | 0-25km | les 2 Tours | 4 | 22.83% | 0.26773 (±0.00611) |
| 19 | 43.34890 | 5.42606 | 0-25km | Collet Redon | 3 | 18.75% | 0.29798 (±0.00506) |
| 20 | 43.34460 | 5.43552 | 0-25km | Curie | 3 | 30.71% | 0.32034 (±0.00582) |
| 21 | 43.34857 | 5.43571 | 0-25km | Nodins | 3 | 21.74% | 0.30478 (±0.00546) |
| 22 | 43.35437 | 5.42971 | 0-25km | Baume | 3 | 32.61% | 0.32806 (±0.00613) |
| 23 | 43.35746 | 5.43290 | 0-25km | Mouret | 3 | 22.83% | 0.30534 (±0.00539) |
| 24 | 43.35433 | 5.44325 | 0-25km | Camoin | 2 | 22.28% | 0.37799 (±0.00518) |
| 25 | 43.36167 | 5.43776 | 0-25km | Parade | 2 | 15.76% | 0.37089 (±0.00491) |
| 26 | 43.35711 | 5.45828 | 0-25km | GraveB | 2 | 19.57% | 0.37843 (±0.00515) |
| 27 | 43.35980 | 5.45991 | 0-25km | Paroyes | 1 | N/A | N/A |
| 28 | 43.29758 | 5.37473 | 0-25km | Vestiges | 1 | N/A | N/A |
| 29 | 43.34015 | 5.44278 | 0-25km | Athena | 6 | 45.65% | 0.22825 (±0.00569) |
| 30 | 43.34331 | 5.40287 | 0-25km | Font Obscure | 7 | 50.27% | 0.22445 (±0.00643) |
| 31 | 43.31491 | 5.42293 | 0-25km | Moline | 4 | 30.43% | 0.26995 (±0.00588) |
| 32 | 43.33292 | 5.44045 | 0-25km | Bégude | 14 | 50.54% | 0.14952 (±0.00575) |
| 33 | 43.30461 | 5.39522 | 0-25km | Longchamps | 16 | 55.16% | 0.14127 (±0.00588) |
| 34 | 43.32530 | 5.41757 | 0-25km | Ginestet | 16 | 63.32% | 0.16565 (±0.0064) |
| 35 | 43.44577 | 5.52859 | 0-25km | Fuveau | 1 | N/A | N/A |
| 36 | 43.60647 | 5.66108 | 25-50km | Saint Antonin2 | 10 | 50.82% | 0.18553 (±0.0064) |
| 37 | 43.70571 | 5.80855 | 50-75km | Boutre | 3 | 13.04% | 0.28202 (±0.00463) |
| 38 | 43.79770 | 5.89677 | 50-75km | Saint Grégoire | 17 | 69.84% | 0.16874 (±0.00648) |
| 39 | 43.86740 | 5.97008 | 75-100km | Valensole | 14 | 62.77% | 0.16821 (±0.00595) |
| 40 | 43.93150 | 6.04693 | 75-100km | Entrevennes | 16 | 66.85% | 0.16359 (±0.00652) |
| 41 | 43.99460 | 6.10607 | 75-100km | Espinouse | 13 | 49.46% | 0.15805 (±0.00627) |
| | | | | **Mean** | 5.3 | 31% | 0.28871 |
