# Supplementary material for: Morphological Clines and Weak Drift along an Urbanization Gradient in the Butterfly, Pieris rapae
Source: PLoS One. 2013 Dec 27;8(12):e83095. doi: 10.1371/journal.pone.0083095 (PMC3873920; doi:10.1371/journal.pone.0083095)
Supplement: Methods S1 — Statistical model formulas and details of implementation in the computing environment R. (PDF) [file pone.0083095.s007.pdf]

Supporting Information

Supporting Methods

Model formulas

Here we describe the environmental correlation models in statistical terms. Each locus (y_i) is tested independently against a single environmental predictor variable (X_i) and tests are evaluated based on significance of the regression coefficient (β or β_1). The mixed models contain intercepts (β_0) and error terms (ε) in addition to the regression coefficient.

Logistic regression:

$yi \;=\; logit - 1(\beta Xi)$

Generalized Estimating Equations:

$yi \;=\; logit - 1(\beta Xi)\;, Var(yi) \;=\; \phi ai$, where the scaling parameter (ϕ) and variance function (a_i) are used to model the covariance structure.

Linear Mixed Model, Geographical Stratification:

$yi \;=\; \beta 0j[i] \;+\; \beta\; 1j[i]\; Xi \;+\; \varepsilon i$, where fixed effects and intercepts vary for *j* geographical groups

Linear Mixed Model, Environmental Stratification:

$yi \;=\; \beta 0j[i] \;+\; \beta 1j[i]\; Xi \;+\; \varepsilon i$, where fixed effects and intercepts vary for *j* environmental classifications

Model details and implementation in R

The environmental correlation models require the following R libraries: *nlme* and *geepack*. To group individuals based on distance class or environmental class you may want to use the function *classIntervals* in the library *classInt* and the function *findCols* in the library *intervals*. Your data should be saved as my.data.frame with columns for the sample site, geographical grouping, environmental grouping, environmental variables, and genotype values at each locus. Each locus and each variable will be tested independently.

Logistic regression was implemented using a binomial error distribution and the logit link function, and significance assessed using an analysis of variance *F*-test. The GEE approach was implemented using autocorrelation correlation structure 1 ('ar1') for sample location and the binomial error distribution, and significance assessed using a Wald test. Under both mixed models, the environmental variable was tested as a fixed effect, with the intercept was allowed to vary for each group. The linear mixed model was implemented with autocorrelation correlation structure 1 ('ar1') and the binomial error distribution. The fixed effect of each variable was compared to a null model without the variable using a likelihood-ratio test to assess significance

Logistic regression:

```
glm.model <- glm(locus ~ variable, family=binomial(link=”logit”))
test.glm <- anova(glm.model, test=”F”)
```

Generalized Estimating Equations:

```
gee.model <- geeglm(locus ~ variable, id=sample.site, data=my.data.frame, corstr=”ar1”,
family=binomial(“logit”))
test.gee <- summary(gee.model)
```

Linear Mixed Model, Geographical Stratification:

```
lmmG.0 <- lme(locus ~ 1, data=my.data.frame, random=1|geographical.grouping, correlation=corAR1(),
method=”ML”)
lmmG.1 <- lme(locus ~ variable, data=my.data.frame, random=1|geographical.grouping,
correlation=corAR1(), method=”ML”)
test.lmmG <- anova(lmmG.0,lmmG.1)
```

Linear Mixed Model, Environmental Stratification:

```
lmmE.0 <- lme(locus ~ 1, data=my.data.frame, random=1|environmental.grouping, correlation=corAR1(),
method=”ML”)
lmmE.1 <- lme(locus ~ variable, data=my.data.frame, random=1|environmental.grouping,
correlation=corAR1(), method=”ML”)
test.lmmE <- anova(lmmE.0,lmmE.1)
```
